# Absence of evidence for increase in risk for autism or attention-deficit hyperactivity disorder following antidepressant exposure during pregnancy: a replication study

**DOI:** 10.1038/tp.2015.190

**Published:** 2016-01-05

**Authors:** V M Castro, S W Kong, C C Clements, R Brady, A J Kaimal, A E Doyle, E B Robinson, S E Churchill, I S Kohane, R H Perlis

**Affiliations:** 1 Department of Psychiatry, Center for Experimental Drugs and Diagnostics, Massachusetts General Hospital, Boston, MA, USA; 2 Partners Research Computing, Partners HealthCare System, One Constitution Center, Boston, MA, USA; 3 Informatics Program, Boston Children's Hospital, Boston, MA, USA; 4 Department of Psychiatry, Psychiatric and Neurodevelopmental Genetics Unit, Massachusetts General Hospital, Boston, MA, USA; 5 Department of Psychiatry, Beth Israel Deaconess Medical Center, Boston, MA, USA; 6 Division of Maternal-Fetal Medicine, Department of Obstetrics and Gynecology, Massachusetts General Hospital, Boston, MA, USA; 7 Analytic and Translational Genomics Unit, Center for Human Genetic Research, Massachusetts General Hospital, Boston, MA, USA; 8 Department of Biomedical Informatics, Harvard Medical School, Boston, MA, USA; 9 Department of Medicine, Brigham and Women's Hospital, Boston, MA, USA

## Abstract

Multiple studies have examined the risk of prenatal antidepressant exposure and risk for autism spectrum disorder (ASD) or attention-deficit hyperactivity disorder (ADHD), with inconsistent results. Precisely estimating such risk, if any, is of great importance in light of the need to balance such risk with the benefit of depression and anxiety treatment. We developed a method to integrate data from multiple New England health systems, matching offspring and maternal health data in electronic health records to characterize diagnoses and medication exposure. Children with ASD or ADHD were matched 1:3 with children without neurodevelopmental disorders. Association between maternal antidepressant exposure and ASD or ADHD liability was examined using logistic regression, adjusting for potential sociodemographic and psychiatric confounding variables. In new cohorts of 1245 ASD cases and 1701 ADHD cases, along with age-, sex- and socioeconomic status matched controls, neither disorder was significantly associated with prenatal antidepressant exposure in crude or adjusted models (adjusted odds ratio 0.90, 95% confidence interval 0.50−1.54 for ASD; 0.97, 95% confidence interval 0.53−1.69 for ADHD). Pre-pregnancy antidepressant exposure significantly increased risk for both disorders. These results suggest that prior reports of association between prenatal antidepressant exposure and neurodevelopmental disease are likely to represent a false-positive finding, which may arise in part through confounding by indication. They further demonstrate the potential to integrate data across electronic health records studies spanning multiple health systems to enable efficient pharmacovigilance investigation.

## Introduction

Prenatal exposure to medications that may disrupt serotonergic neurotransmission has been proposed as an environmental risk factor for autism, motivated largely by rodent studies demonstrating an impact of such disruption on subsequent behavior.^1, 2^ In multiple investigations based on registries, electronic health records (EHRs), or claims databases, some indication of increased risk following antidepressant exposure was identified, initially for autism spectrum disorder (ASD) and later for attention-deficit hyperactivity disorder (ADHD), although the extent to which such risk represented confounding by indication could not be confidently determined,^3, 4^ and a recent large-scale Danish registry-based study did not replicate the ASD association.^5^

Likewise, in a previous EHR study in a large health system, we did not detect elevated autism risk after controlling for aspects of maternal psychopathology.^6^ However, elevated ADHD risk—without clear mechanistic explanation—was detected and not fully explained by maternal psychiatric illness.

The precise estimation of risk, if any, has substantial public health implications. The decision to expose a developing fetus to any medication is a difficult one for parents and clinicians; such a decision may be even more fraught in case of depression and related disorders, where the potential benefits of treatment are still misunderstood by the lay press^7^ and clinicians despite evidence that treatment discontinuation increases depressive recurrence risk substantially.^8^ Therefore, we extended our methods to allow matching of children and mothers across different health systems without exposing identifying information, and applied this approach to identify another large, independent cohort of children with ASD and ADHD and matched controls for a replication study.

## Materials and methods

### Overview and data set generation

To maximize comparability with prior work, cohort definitions and analytic strategies followed those described in our prior report.^6^ Sociodemographic and clinical data were drawn from three independent EHRs: the Partners HealthCare system, which spans Massachusetts General Hospital (MGH), Brigham and Women's Hospital and Newton-Wellesley Hospital, as well as affiliated outpatient clinics; the Beth Israel Deaconess Medical Center (BIDMC); and the Boston Children's Hospital. Additional maternal and paternal data, as well as confirmation of matching accuracy between mothers and offspring were obtained from the Massachusetts Registry of Vital Records and Statistics.

Specific data drawn from EHRs included sociodemographic data, billing codes, problem lists and medications. These data were managed with the i2b2 server software (i2b2 v1.6.04, Boston, MA, USA),^9, 10, 11^ a scalable computational framework for managing human health data.

Mothers were identified on the basis of matching child's date of birth and surname, insurance identifiers and hospital encounter date. As a further confirmation of match, and to address cases where children might have different last names or where they might have been removed from maternal custody, Massachusetts state birth certificates were queried for all identified children. Where the mother−child matches could not be confirmed, those pairs were omitted from analysis. Consistent with prior reports, we restricted the analysis to one child per mother, choosing the child with ASD or ADHD when a mother had both a case and control offspring. When two case or two control children were identified from one mother we randomly selected one child for inclusion in the study. Matching across, institutions used a one-way hash of maternal and child identifiers;^12^ this allowed unique individuals to be matched without revealing names or other identifiers outside a given health system. For example, investigators in the Partners HealthCare system never received access to identifiers from the Children's Hospital system, and vice versa. Once the linking was complete, each mother and child was assigned a study-specific identifier and all identifiers were removed from the analysis file.

The Institutional Review Boards of Partners HealthCare, BIDMC and Boston Children's Hospital approved all aspects of this study. Access to Massachusetts birth certificates was approved by the Massachusetts Department of Public Health Institutional Review Board.

### Cohort definition

Eligible children for the ASD cohort were identified from the Partners and Boston Children's Hospital EHRs if they met the following criteria: age 2−19 years; at least one ICD-9 code of 299 (pervasive developmental disorder) between 1997 and 2010; delivered at MGH, Brigham and Women's Hospital, Newton-Wellesley Hospital or BIDMC. The ADHD cohort comprised children age 2−19 years with at least one ICD-9 code of 314.x and no ICD-9 code of 299 between 1997 and 2010, delivered at MGH, Brigham and Women's Hospital, Newton-Wellesley Hospital or BIDMC. Prior work demonstrated that these ICD-9 codes have a high sensitivity and specificity for ASD and ADHD versus generally healthy control children (that is, those without diagnosis or symptoms of ASD or ADHD) in the Partners Healthcare system based on review of 50 ASD, ADHD and control records by an experienced child neuropsychologist,^6^ as other reports also suggested.^3^ Mother−child pairs included in our previous report were excluded.

These children were then matched 1:3 with non-ASD control children delivered at MGH, Brigham and Women's Hospital, Newton-Wellesley Hospital or BIDMC with the same year of birth, birth hospital, sex, insurance type as a proxy for socioeconomic status, race/ethnicity and preterm versus full-term status. Children with any history of ASD, ADHD or intellectual disability (ICD-9 of 299, 314 or 317−319) were excluded from the control population. If fewer than three matches could be identified for a case, year of birth was relaxed so that controls were born within 3 years of a given case. Sixty-five ASD cases (2.5%) had no matches among healthy control children and were excluded from primary analysis; their inclusion in exploratory analysis did not change results; 107 ASD cases matched fewer than 3 controls but were included in the analysis with 1 or 2 matched controls. In all, 138 ADHD cases (3.5%) had no matches among healthy control children and were excluded from primary analysis; their inclusion in exploratory analysis did not change results; 317 ADHD cases matched fewer than 3 controls but were included in the analysis with 1 or 2 matched controls.

### Exposure definition

We used the definitions of exposure applied in prior work.^3, 6^ Primary analyses examined any exposure during pregnancy (from the 90 days before last menstrual period (LMP) through delivery) and before pregnancy (any time before LMP). Additional exposure windows were calculated based on estimated LMP based on gestational age—this included preconception (3 months before LMP), first trimester (0−90 days following LMP), second trimester (91−180 days following LMP) and third trimester (>180 days after LMP up to delivery). Exposures were identified using e-prescribing data in the EHR, both inpatient and outpatient, which record number of pills, frequency and refill number, allowing calculation of exposure period. Even where the clinician is not the primary prescriber (for example, for patients receiving psychiatric medications outside of this health system), hospital policy requires reconciliation of medication lists at outpatient visits and hospital admission. The exposure period was truncated when discontinuation was recorded by the clinician. Exposure was defined formally as overlap between a medication prescription window and a given time period (entire pregnancy or trimester, for example) By prior agreement between the Partners HealthCare system and Surescript, medication dispensation data is not allowed to be applied for research purposes and so is unavailable.

### Analysis

Three sets of models were examined for association between individual baseline variables and ASD or ADHD versus control status. In crude analyses, simple conditional logistic regression estimated the odds ratios for disease based on predictor. In model 1, conditional logistic regression models also included gender, race, year of birth, type of maternal insurance (public versus private) and median maternal income derived from census tract; these variables were selected *a priori* as potential confounding variables and to ensure consistency with our prior report.^6^ In model 2, presence or absence of maternal major depressive disorder (ICD-9 code of 296.2x or 296.3x) was added to model 1, addressing confounding by indication. Adjusted odds ratios were calculated for both model 1 and 2.

In light of concern for confounding by maternal psychopathology raised in prior reports, proxies for maternal illness severity, including number of psychopharmacologic visits, number of psychotherapy visits, different antidepressant medications in the prior year, and specific psychiatric disorder or comorbidity were also examined in regression models.

All analyses used R 3.0.1 (The R Foundation for Statistical Computing, Vienna, Austria).

## Results

Table 1 summarizes the baseline characteristics of the ASD and ADHD cohorts, as well as the healthy controls matched 3:1 with each case group. Table 1 also reports crude and adjusted odds ratios for association between individual characteristics and disease status. As in our prior report, greater maternal age was associated with increased ASD risk, while younger maternal age was associated with increased ADHD risk. For both diagnoses, greater maternal (and paternal) education was associated with decreased risk in offspring. Finally, for ADHD but not ASD, offspring of mothers with public versus private insurance were at elevated risk.

We next examined ASD and ADHD risk associated with antidepressant exposure before and during pregnancy (Table 2). For both ASD and ADHD, pre-pregnancy antidepressant use was associated with greater risk, even after adjustment for maternal major depression. However, no significant increase in risk during pregnancy as a whole, or with exposure during particular trimesters, was observed for either disorder. To allow comparison with prior reports, high and low/moderate-affinity antidepressants were examined separately in regression models. Neither group yielded significant associations with ASD or ADHD risk (Supplementary Table 1).

Finally, to elucidate the potential confounding effects of illness severity, Table 3 summarizes features of maternal illness extracted from the EHR. We observed that maternal psychotherapy appears to be associated with a significant increase in ASD risk. Estimates of risk for ASD or ADHD associated with individual maternal diagnoses varied widely, suggesting the complexity of the familial relationship between childhood neurodevelopmental disorders and maternal psychiatric illness, and reflecting the modest numbers of individuals represented despite the relatively large case and control cohorts. However, incorporating individual terms in risk models for ASD or ADHD did not meaningfully change results: in no case was the resulting adjusted odds ratio changed by >10%, nor was it significantly different from 1.

## Discussion

In an effort to clarify the risk for neurodevelopmental disorders associated with prenatal antidepressant exposure, we applied a novel methodology to identify a new and independent cohort of ASD cases, another of ADHD cases, and matched cohorts of healthy control children. Data were drawn from three large Boston-based health systems as well as state birth certificate records. For both ASD and ADHD, we find no significant increase in risk associated with prenatal antidepressant exposure. Supporting the possibility that prior reports represent confounding by indication, we do identify risk associated with pre-pregnancy antidepressant exposure, and with prenatal maternal psychotherapy. In other words, the requirement for maternal antidepressant treatment, rather than the medication itself, may be associated with risk for neurodevelopmental disorders in offspring.

We recognize that even estimates of risk derived from large cohorts still represent only estimates. So, for example, we can only exclude an increase in odds of ASD >54% with 95% confidence interval. (By the same token, an equally likely prospect is a protective effect of the same magnitude). In all cases, the risk of a prescription for a given medication must be weighed against the potential benefits. In aggregate, the present results should in general increase confidence that any treatment-associated risk is likely to be quite modest. Still, in light of recent media portrayals of depression during pregnancy^7^ equating antidepressant use with smoking, it is clear that, for some individuals, any risk would be considered to be too great, perhaps because of an unwillingness to recognize the potential neonatal morbidity as well as the known maternal morbidity and mortality associated with depression and related disorders. In our prior report, we found evidence of ADHD risk that persisted despite adjustment for maternal psychopathology. We suggested at that time that, given the risk of that finding representing a false positive, further study was warranted.^6^ The present results suggest the importance of such follow-up, indicating that ADHD risk is unlikely to be substantially increased by antidepressant exposure. We also note that, unlike ASD where a rodent model initially posited risk,^6^ the prior probability for association between ADHD and antidepressant exposure would be lower in the absence of a clear mechanism of risk.

We could identify no reason for the discordance between this result and our prior one with regard to ADHD. Of note, the non-antidepressant risk factors for ASD and ADHD (for example, maternal age and socioeconomic status) are still noted in the present cohort, suggesting that in other respects it is similar to those in prior descriptions. Likewise, while antidepressant prescribing rates are somewhat lower (primarily because of the inclusion of another hospital with lower antidepressant prescribing rates), the variables associated with such prescriptions remain similar to those in our prior report. Incorporating terms for delivery site, or diagnosis site, did not meaningfully impact results.

The present study has multiple limitations. First, as already noted, we cannot exclude more modest risk, which would require substantially greater sample sizes. Broader application of the cross-institution approach we describe may represent; first, means of generating such cohorts. Second, there persists some risk for confounding, as any proxy of maternal psychopathology other than direct measurement is imperfect. On the other hand, in light of evidence of efficacy for depression treatment during pregnancy, a randomized trial might be challenged on ethical grounds. Third, there is some risk of misclassification among control subjects, if ASD or ADHD was diagnosed at another institution, which would tend to decrease power to detect true association. The relatively low prevalence of ASD should minimize the impact of such misclassification; in contrast to most prior studies, by spanning the two primary ASD referral centers (MGH and Boston Children's Hospital) in this region, the probability of such misidentification is substantially diminished. Moreover, while we used a case definition previously validated by chart review that included scores from autism diagnostic observation scale,^13^ some misclassification of cases may also diminish power.

In the context of prior reports, this study illustrates both the power and the challenge of *in silico* pharmacovigilance studies. Without recruitment of a single individual, existing EHR data facilitated generation of a very large cohort of at-risk patients, matching mothers and offspring. On the other hand, such nonrandomized data presents a high risk for confounding, even when EHRs with more detailed health data are available for analysis. We also note that, while the data generation and analysis required ~4 weeks, interactions with multiple institutional review boards and development of protocols acceptable to these independent bodies required nearly 2 years. Thus, while it is not reflected directly in our results, we would argue that this study also highlights the need for more consistent review policies and protocols across institutions.

Taken together, our results in this new cohort provide some clarification of the magnitude of risk reported in prior investigations. Paired with estimates of the benefit of treating depression and related disorders during and after pregnancy, they should aid patients and clinicians in decision-making about this class of psychotropic medications. More broadly, these results illustrate a strategy for linking across health systems to study rarer exposures or outcomes.

## Figures and Tables

**Table 1:** Sociodemographic and medical history of children and parents in ASD, ADHD and ASD-matched control study groups

	*ASD*	*ASD controls*	*ASD versus matched controls*		*ADHD*	*ADHD controls*	*ADHD versus matched controls*	
*N*=	*1245*	*3405*	*Unadjusted*	*Model 1*	*1701*	*3797*	*Unadjusted*	*Model 1*
			*OR (95% CI)*	*OR (95% CI)*			*OR (95% CI)*	*OR (95% CI)*
*Child demographics*								
Age at first diagnosis, years (mean (s.d.))	5.5 (3.3)	—	—	—	8.8 (2.9)		—	—
Male gender	78.9%	79.6%	0.96 (0.82−1.12)	0.95 (0.79−1.14)	75.8%	79.6%	0.80 (0.70−0.92)*	0.76 (0.65−0.89)*
Race								
White	65.2%	68.7%	0.85 (0.74−0.98)*	0.74 (0.57−0.97)*	73.4%	78.1%	0.77 (0.68−0.88)*	0.74 (0.57−0.95)*
Black	10.9%	9.0%	1.24 (1.00−1.53)*	1.53 (1.14−2.03)*	9.6%	7.2%	1.37 (1.11−1.67)*	1.51 (1.15−1.96)*
Hispanic	6.7%	5.8%	1.16 (0.88−1.50)	1.51 (1.05−2.16)*	6.6%	5.0%	1.33 (1.04−1.69)*	1.58 (1.15−2.17)*
Asian	7.3%	7.4%	0.97 (0.76−1.25)	1.21 (0.90−1.62)	1.9%	2.5%	0.76 (0.50−1.12)	0.74 (0.45−1.15)
Other	10.0%	9.0%	1.11 (0.89−1.38)	1.36 (1.04−1.77)*	8.5%	7.1%	1.21 (0.98−1.50)	1.36 (1.05−1.74)*
* *Median household income								
First tertile (< $32,136)	29.3%	34.2%	0.80 (0.68−0.93)*	0.92 (0.74−1.14)	31.5%	33.7%	0.91 (0.79−1.04)	0.93 (0.77−1.12)
Second tertile ($32,136−$41,820)	38.3%	31.2%	1.37 (1.17−1.59)*	1.49 (1.22−1.82)*	37.4%	31.1%	1.33 (1.16−1.51)*	1.42 (1.19−1.69)*
Third tertile (> $41,820)	32.4%	34.5%	0.91 (0.78−1.06)	1.09 (0.88−1.35)	31.1%	35.2%	0.83 (0.72−0.95)*	1.08 (0.90−1.30)
Multiple birth	10.7%	8.4%	1.31 (1.05−1.62)*	1.28 (0.98−1.65)	6.9%	8.0%	0.85 (0.68−1.06)	0.96 (0.74−1.24)
								
*Parent demographics*								
Mother age at delivery, years (mean (s.d.))	32.4 (5.4)	31.8 (5.6)	1.02 (1.01−1.03)*	1.04 (1.02−1.05)*	31.1 (6.2)	32.0 (5.4)	0.97 (0.96−0.98)*	0.99 (0.98−1.01)
Father age at delivery, years (mean (s.d.))	34.9 (6.5)	34.4 (6.2)	1.01 (1.00−1.02)*	1.00 (0.98−1.02)	33.3 (6.8)	34.4 (6.0)	0.97 (0.96−0.98)*	0.99 (0.97−1.00)
Mother−government insurance	16.3%	14.9%	1.11 (0.93−1.32)	1.20 (0.94−1.53)	18.6%	14.0%	1.41 (1.21−1.64)*	1.31 (1.06−1.62)*
Mother, 4+ years of college	63.7%	68.5%	0.81 (0.70−0.92)*	0.83 (0.69−1.01)	60.0%	69.7%	0.65 (0.58−0.73)*	0.82 (0.69−0.97)*
Father, 4+ years of college	64.8%	69.8%	0.80 (0.69−0.92)*	0.78 (0.64−0.94)*	58.4%	70.8%	0.58 (0.51−0.66)*	0.61 (0.52−0.72)*
								
*Mother past medical history*								
Diabetes mellitus	4.1%	2.2%	1.91 (1.32−2.73)*	2.21 (1.45−3.35)*	3.6%	2.0%	1.82 (1.29−2.56)*	2.28 (1.53−3.38)*
Chronic hypertension	3.1%	2.1%	1.53 (1.02−2.26)*	1.17 (0.69−1.92)	2.9%	2.0%	1.52 (1.05−2.18)*	1.95 (1.29−2.92)*
Major depressive disorder	7.8%	7.7%	1.01 (0.79−1.29)	1.21 (0.87−1.66)	4.7%	4.8%	0.99 (0.75−1.29)	1.06 (0.75−1.49)
* *Parity								
1	57.7%	52.6%	1.23 (1.08−1.41)*	1.30 (1.11−1.53)*	53.4%	52.4%	1.04 (0.92−1.17)	1.05 (0.92−1.21)
2	33.1%	34.8%	0.93 (0.81−1.07)	0.93 (0.79−1.09)	35.3%	34.9%	1.01 (0.90−1.15)	0.99 (0.85−1.14)
3+	9.2%	12.6%	0.70 (0.56−0.87)*	0.62 (0.48−0.80)*	11.4%	12.6%	0.89 (0.74−1.06)	0.91 (0.74−1.13)

Abbreviations: ASD, autism spectrum disorder; ADHD, attention-deficit hyperactivity disorder; CI, confidence interval; OR, odds ratio.

*Indicates uncorrected *P*<0.05.

**Table 2:** Maternal antidepressant exposure before and during pregnancy associated with risk for (a) ASD and (b) ADHD

(a)					
*Antidepressant exposure*	*ASD*	*Controls*	*ASD* versus *ASD-matched controls^a^*		
	*1245 (%)*	*3405 (%)*	*Unadjusted*	*Model 1*	*Model 2*
			*OR (95% CI)*	*OR (95% CI)*	*OR (95% CI)*
*Time period*					
Prepregnancy	4.6	3.6	1.29 (0.93−1.77)	1.63 (1.02−2.54)*	1.54 (1.02−2.30)*
Pregnancy (preconception delivery)	2.2	2.3	0.99 (0.63−1.51)	1.13 (0.61−2.00)	0.90 (0.50−1.54)
Preconception (conception−30 days)	1.0	0.9	1.14 (0.57−2.14)	1.62 (0.62−3.79)	1.12 (0.46−2.47)
First trimester	1.4	1.3	1.07 (0.59−1.86)	1.10 (0.45−2.41)	0.89 (0.40−1.78)
Second trimester	1.3	1.1	1.18 (0.63−2.08)	1.39 (0.59−2.99)	1.11 (0.50−2.26)
Third trimester	1.4	1.4	1.00 (0.56−1.72)	1.20 (0.53−2.47)	0.85 (0.38−1.74)

(b)					
*Antidepressant exposure*	*ADHD*	*Controls*	*ADHD* versus *ADHD-matched controls^a^*		
	*1701 (%)*	*3797 (%)*	*Unadjusted*	*Model 1*	*Model 2*
			*OR (95% CI)*	*OR (95% CI)*	*OR (95% CI)*
*Time period*					
Prepregnancy	3.1	3.8	1.22 (0.89−1.66)	1.48 (1.01−2.15)*	1.50 (1.00−2.20)*
Pregnancy (preconception delivery)	1.7	1.5	0.91 (0.56−1.42)	1.00 (0.56−1.71)	0.97 (0.53−1.69)
Preconception (conception−30 days)	0.8	0.7	0.92 (0.45−1.77)	1.04 (0.43−2.29)	1.01 (0.41−2.26)
First trimester	1.1	0.7	0.67 (0.33−1.24)	0.72 (0.31−1.52)	0.69 (0.29−1.49)
Second trimester	0.9	0.8	0.89 (0.46−1.63)	1.13 (0.51−2.28)	1.11 (0.50−2.26)
Third trimester	1.0	0.6	0.64 (0.31−1.22)	0.75 (0.30−1.65)	0.73 (0.29−1.62)

Abbreviations: ASD, autism spectrum disorder; ADHD, attention-deficit hyperactivity disorder; CI, confidence interval; OR, odds ratio. ^a^Unadjusted and adjusted risk of ADHD and ASD compared with ADHD- and ASD-matched controls respectively; Significance at *P*<0.05. Model 1 is adjusted for gender, race, birth year, insurance type, and median income tertile. Model 2 is adjusted for variables in Model 1 and past history of maternal depression.

**Table 3:** Association between maternal psychiatric morbidity and risk for (a) ASD or (b) ADHD

(a)				
*Depression severity measure*		*ASD*	*Controls*	*ASD* versus *ASD-matched controls*
		*1245 (%)*	*3405 (%)*	*Adjusted model 1*
	*Timeframe*			*Severity OR (95% CI)*
*Mental health treatments*				
Distinct antidepressants (two or more)	Within 2 years before delivery	0.6	0.6	0.96 (0.27−2.70)
Psychotherapy visits (one or more)	Pregnancy	3.4	1.4	2.40 (1.46−3.90)*
Psychotherapy visits (two or more)	Pregnancy	1.7	0.8	2.09 (1.07−3.96)*
Psychopharmacolgy visits (one or more)	Pregnancy	0.6	0.5	1.62 (0.61−3.91)
				
*Psychiatric history*				
Bipolar isorder	Before delivery	0.6	0.6	0.87 (0.28-2.19)
History of substance/alcohol abuse	Before delivery	3.4	3.1	1.01 (0.63−1.56)
Past suicide attempt	Before delivery	0.3	0.3	1.37 (0.29−5.07)
Schizophrenia or schizoaffective disorder	Before delivery	0.2	0.1	0.49 (0.03−3.08)
PTSD	Before delivery	1.0	0.3	2.58 (0.97−6.61)*
OCD	Before delivery	0.3	0.2	1.05 (0.22−3.73)
GAD	Before delivery	0.7	0.3	3.27 (1.12−9.35)*
Depressive disorder (ICD-9 311)	Before delivery	5.7	4.8	1.32 (0.93−1.84)
Panic disorder	Before delivery	0.8	0.8	1.17 (0.51−2.48)
Any psychiatric history of the above or MDD	Before delivery	14.0	13.7	1.12 (0.88−1.42)

(b)				
*Depression severity measure*		*ADHD*	*Controls*	*ADHD* versus *ADHD-matched controls*
		*1701 (%)*	*3797 (%)*	*Adjusted model 1*
	*Timeframe*			*Severity OR (95% CI)*
*Mental health treatments*				
Distinct antidepressants (two or more)	Within 2 years before delivery	0.5	0.3	1.94 (0.70−5.04)
Psychotherapy visits (one or more)	Pregnancy	3.3	1.4	1.55 (0.97−2.45)
Psychotherapy visits (two or more)	Pregnancy	1.8	0.7	1.69 (0.89−3.14)
Psychopharmacolgy visits (one or more)	Pregnancy	0.5	0.4	1.58 (0.58−3.96)
				
*Psychiatric history*				
Bipolar disorder	Before delivery	0.9	0.6	1.77 (0.80−3.73)
History of substance/alcohol abuse	Before delivery	4.5	2.7	1.57 (1.09−2.24)*
Past suicide attempt	Before delivery	0.4	0.2	3.30 (0.85−12.72)
Schizophrenia or schizoaffective disorder	Before delivery	0.2	0.2	1.05 (0.21−4.28)
PTSD	Before delivery	0.6	0.5	0.75 (0.24−2.00)
OCD	Before delivery	0.2	0.2	0.65 (0.10−2.69)
GAD	Before delivery	0.4	0.2	2.59 (0.82−7.96)
Depressive disorder (ICD-9 311)	Before delivery	5.6	4.3	1.44 (1.05−1.95)*
Panic disorder	Before delivery	1.0	0.7	1.13 (0.52−2.31)
Any psychiatric history of the above or MDD	Before delivery	12.6	10.0	1.42 (1.14−1.76)**

Abbreviations: ASD, autism spectrum disorder; ADHD, attention-deficit hyperactivity disorder; CI, confidence interval; GAD, generalized anxiety disorder; MDD, major depressive disorder; OCD, obsessive compulsive disorder; OR, odds ratio; PTSD, post-traumatic stress disorder. *Indicates uncorrected *P*<0.05. **Indicates uncorrected *P*<0.01.
