# Supplementary material for: Absence of evidence for increase in risk for autism or attention-deficit hyperactivity disorder following antidepressant exposure during pregnancy: a replication study
Source: Transl Psychiatry. 2016 Jan 5;6(1):e708–. doi: 10.1038/tp.2015.190 (PMC5068870; doi:10.1038/tp.2015.190)
Supplement: Supplementary Table 1 [file tp2015190x1.doc]

Supplemental Table 1. Maternal antidepressant exposure prior to and during pregnancy associated with risk for a) ASD and b) ADHD by serotonin transporter affinity.

a)

|  | **ASD** | **Controls** | **ASD vs. ASD-matched Controls** | | | |
| --- | --- | --- | --- | --- | --- | --- |
|  | 1,245 | 3,405 | Unadjusted | | Model 2 | |
| **High 5-HT Antidepressant exposure** |  |  | OR (95% CI) | | OR (95% CI) | |
| Time period |  |  |  |  |  |  |
| Prepregnancy | 2.8% | 2.1% | 1.33 (0.87-1.99) |  | 1.34 (0.87-2.05) |  |
| Pregnancy (preconception - delivery) | 1.5% | 1.5% | 1.01 (0.58-1.69) |  | 1.01 (0.57-1.73) |  |
| Preconception (conception - 30 days) | 0.7% | 0.5% | 1.44 (0.61-3.17) |  | 1.50 (0.63-3.37) |  |
| 1st Trimester | 0.9% | 0.8% | 1.15 (0.54-2.27) |  | 1.12 (0.52-2.27) |  |
| 2nd Trimester | 0.8% | 0.7% | 1.18 (0.54-2.42) |  | 1.26 (0.56-2.64) |  |
| 3rd Trimester | 0.8% | 0.9% | 0.87 (0.41-1.73) |  | 0.96 (0.44-1.93) |  |
|  | **ASD** | **Controls** | **ASD vs. ASD-matched Controls** | | | |
|  | 1,245 | 3,405 | Unadjusted | | Model 2 | |
| **Low/Moderate 5-HT antidepressant exposure** |  |  | OR (95% CI) | | OR (95% CI) | |
| Time period |  |  |  |  |  |  |
| Prepregnancy | 2.5% | 2.2% | 1.11 (0.72-1.68) |  | 1.16 (0.74-1.79) |  |
| Pregnancy (preconception - delivery) | 1.0% | 0.9% | 1.02 (0.50-1.93) |  | 0.95 (0.46-1.84) |  |
| Preconception (conception - 30 days) | 0.3% | 0.4% | 0.72 (0.21-2.00) |  | 0.64 (0.18-1.81) |  |
| 1st Trimester | 0.5% | 0.5% | 0.96 (0.35-2.31) |  | 0.84 (0.30-2.08) |  |
| 2nd Trimester | 0.5% | 0.5% | 1.02 (0.36-2.48) |  | 1.01 (0.35-2.52) |  |
| 3rd Trimester | 0.8% | 0.6% | 1.43 (0.64-3.03) |  | 1.30 (0.57-2.83) |  |
| b) |  |  |  |  |  |  |
|  |  |  |  |  |  |  |
|  | **ADHD** | **Controls** | **ADHD vs. ADHD-matched Controls** | | | |
|  | 1,701 | 3,797 | Unadjusted | | Model 2 | |
| **High 5-HT antidepressant exposure** | **%** | % | OR (95% CI) | | OR (95% CI) | |
| Time period |  |  |  |  |  |  |
| Prepregnancy | 2.2% | 1.7% | 1.26 (0.83-1.88) |  | 1.49 (0.95-2.29) |  |
| Pregnancy (preconception - delivery) | 0.8% | 1.2% | 0.71 (0.37-1.26) |  | 0.64 (0.33-1.19) |  |
| Preconception (conception - 30 days) | 0.3% | 0.5% | 0.62 (0.20-1.55) |  | 0.63 (0.20-1.65) |  |
| 1st Trimester | 0.3% | 0.7% | 0.44 (0.15-1.07) |  | 0.45 (0.15-1.12) |  |
| 2nd Trimester | 0.4% | 0.6% | 0.64 (0.23-1.49) |  | 0.60 (0.21-1.45) |  |
| 3rd Trimester | 0.4% | 0.6% | 0.65 (0.26-1.43) |  | 0.61 (0.24-1.41) |  |
|  | **ADHD** | **Controls** | **ADHD vs. ADHD-matched Controls** | | | |
|  | 1,701 | 3,797 | Unadjusted | | Model 2 | |
| **Low/Moderate 5-HT antidepressant exposure** | **%** | % | OR (95% CI) | | OR (95% CI) | |
| Time period |  |  |  |  |  |  |
| Prepregnancy | 1.9% | 1.9% | 0.98 (0.63-1.47) |  | 1.18 (0.75-1.82) |  |
| Pregnancy (preconception - delivery) | 0.8% | 0.6% | 1.21 (0.60-2.35) |  | 1.39 (0.67-2.76) |  |
| Preconception (conception - 30 days) | 0.4% | 0.3% | 1.30 (0.48-3.25) |  | 1.45 (0.51-3.80) |  |
| 1st Trimester | 0.4% | 0.4% | 1.04 (0.40-2.47) |  | 1.15 (0.42-2.86) |  |
| 2nd Trimester | 0.5% | 0.4% | 1.19 (0.48-2.75) |  | 1.51 (0.59-3.57) |  |
| 3rd Trimester | 0.3% | 0.4% | 0.70 (0.23-1.78) |  | 0.89 (0.28-2.33) |  |
